# Valorization of Lignin by Partial Wet Oxidation Using Sustainable Heteropoly Acid Catalysts

**DOI:** 10.3390/molecules22101625

**Published:** 2017-09-28

**Authors:** Abayneh Getachew Demesa, Arto Laari, Mika Sillanpää, Tuomas Koiranen

**Affiliations:** 1 Laboratory of Process and Product Development, LUT School of Engineering Science, Lappeenranta University of Technology, Skinnarilankatu 34, FI-53850 Lappeenranta, Finland; arto.laari@lut.fi (A.L.); tuomas.koiranen@lut.fi (T.K.); 2 Laboratory of Green Chemistry, LUT School of Engineering Science, Lappeenranta University of Technology, Sammonkatu 12, FI-50130 Mikkeli, Finland; mika.sillanpaa@lut.fi

**Keywords:** catalysts, heteropoly acids, lignin, wet oxidation

## Abstract

The production of carboxylic acids by partial wet oxidation of alkali lignin at elevated temperatures and pressures was studied experimentally. Two different heteropoly acids, phosphotungstic acid (H_3_PW_12_O_40_) and phosphomolybdic acid (H_3_PMo_12_O_40_), were used to catalyze the oxidation of lignin under hydrothermal conditions. Factors influencing the total yield of carboxylic acids formed during the partial oxidation of lignin were investigated. Formic, acetic and succinic acids were the major products identified. Of the two catalysts used, phosphomolybdic acid gave the most promising results, with carboxylic acid yields and lignin conversions of up to 45% and 95%, respectively.

## 1. Introduction

Industrial production of a wide range of chemicals and fuels relies greatly on fossil resources. However, uncertainty surrounding oil prices and dwindling reserves of easily extractable fossil fuels, along with growing environmental concerns, have resulted in increased attention being given to the possible replacement of existing fossil-based feedstocks with renewable resources [1]. To meet the growing demand for renewable fuels, biomass, which is considered a sustainable source of organic carbon [2,3], has been proposed as an alternative to petroleum for the production of fuels and fine chemicals with net zero carbon emissions [4,5]. In this respect, lignocellulosic biomass, which is the most abundant and sustainable biomass on earth, has great importance [4]. In addition, lignocellulosic feedstocks have significant advantages over other biomass sources since they do not directly interfere with the food chain, as they utilize non-edible portions of the plant [6], and from an economic perspective, lignocellulosic biomass can be produced quickly and at a lower cost than agriculturally important biofuel feedstocks, such as soybeans, starch, and sugarcane [7,8]. The efficient utilization of lignocellulosic biomass has been the focus of considerable research and development efforts, with the aim of replacing petroleum-based products with an environmentally sustainable alternative [7].

Lignin, the second most abundant natural polymer, has potential as a promising alternative to fossil resources, for the sustainable production of chemicals and fuels, to reduce CO_2_ emissions and hence global warming. However, compared to cellulose, lignin has received little attention, in regards to valorization; hence, its potential has hitherto remained underutilized [9]. The primary reason for the limited interest is that the heterogeneous and cross-linked structure of lignin molecules complicates the controlled depolymerization of lignin into single chemicals [10,11]. Yet, lignin holds promise as a significant resource for the production of a wide range of renewable chemicals, due to its high energy content, aromatic structure, the presence of highly reactive groups, and the fact that it is generated in large volumes when second generation biorefineries are deployed [12].

Different methods for converting lignocellulose into high-value products are discussed in the literature, including gasification, pyrolysis, solvolysis, hydrogenolysis, hydrolysis and oxidation [10,11]. Partial wet oxidation by molecular oxygen has been proposed as a potential process for the production of chemicals from biomass wastes [11]. Wet oxidation is especially well suited for the treatment of low-value, water-containing biomass that is difficult or expensive to treat by other means. In the partial wet oxidation process, organic compounds are oxidized at elevated temperatures (125–320 °C) and pressures (0.5–2 MPa), using gaseous oxygen or air as the oxidant [11]. The high pressure in partial wet oxidation helps to maintain the liquid phase, which in turn increases the concentration of dissolved oxygen and thus, the oxidation rate. The wet oxidation process has the further benefits of using relatively cheap molecular oxygen and not requiring other harmful chemical reagents [13].

Partial wet oxidation of biomass can be carried out either catalytically or non-catalytically. Non-catalytic partial wet oxidation of lignin in an alkaline condition to produce carboxylic acids has been previously studied in [10], where formic acid, acetic acid, succinic acid, oxalic acid and glutaconic acid were the main products identified. These compounds are not only important commodity chemicals in themselves, but are also intermediates in the production of various fuels and chemicals through further transformations. It was also found that operational conditions such as reaction time, temperature and initial lignin concentration greatly affected the yield of products. Moreover, the total yield of products was found to be reduced at higher initial lignin concentrations, which was attributed to repolymerization/condensation side reactions of lignin fragments that compete with oxidative lignin depolymerization reactions, as indicated in measured molecular weight distributions.

The catalytic conversion of biomass is a very complex process that usually involves several reaction pathways taking place in parallel or successively depending on specific precursors, reaction conditions and catalysts [14]. However, catalytic biomass conversion is often preferred since it provides a cost-effective and environmentally benign route to the desired products, with a high reaction rate and selectivity [14]. Various types of catalysts, from heterogeneous noble metal catalysts [14,15,16], to inexpensive homogeneous metal ion catalysts [17,18,19,20,21], such as iron, copper, and cobalt have been extensively studied for lignin catalytic oxidation processes. Noble metals are however, expensive, which affects their commercial viability, whereas homogeneous catalysts, such as cupric sulfate, are difficult to separate from the reaction mixture, which can lead to secondary pollution, resulting in high recycling costs after use [22]. Heterogeneous catalysts, on the other hand, can be easily separated and regenerated [23,24,25]. However, it can be expected that in the treatment of solid biomass, they are prone to low catalytic activity, due to poor access for solids on the catalyst surface. It is, therefore, important to find suitable homogenous catalysts that are readily separable and reusable after partial wet oxidation and that have the lowest possible environmental impact.

Heteropoly acids (HPAs) are active catalysts for both homogeneous and heterogeneous acid-catalyzed reactions, and some of them are also strong oxidants. HPAs are considered environmentally benign and economically feasible solid catalysts, due to their excellent functional characteristics, such as ease of handling and removal, reusability, few side reactions, strong Brønsted acidity approaching the super-acid region, and high proton mobility, stability and catalytic activity [26,27]. Because of their high solubility in water, HPAs are more efficient for catalyzing reactions in biomass than water-insoluble solid acid catalysts. Moreover, solid heteropoly acids are insoluble in non-polar hydrocarbons, which makes it possible to recover them from the reaction mixture, without the need for neutralization, simply by extraction or precipitation [28,29]. The potential of heterogeneous or immobilized HPAs [30,31] and homogeneous HPAs [32,33,34,35,36,37] as catalysts for biomass conversion has been widely studied. However, most of the studies used cellulose as the substrate and in many instances, had fixed operational conditions, thus they provide no indication of optimal parameters. Consequently, to determine the best possible reaction conditions, more studies are needed, covering a wider range of operational conditions.

In this research, lignin is used as the feed material, and the catalytic potential of two homogenous heteropoly acid catalysts—phosphotungstic acid (H_3_PW_12_O_40_) and phosphomolybdic acid (H_3_PMo_12_O_40_)—for the partial wet oxidation of lignin are evaluated, based on their effectiveness in regards to carboxylic acid yield and lignin conversion. The influence of different operational conditions is also investigated, to determine the optimal conditions to achieve the maximum yield of products and lignin conversion.

## 2. Results and Discussion

Formic, acetic, and succinic acids were the main carboxylic acid compounds identified by HPLC, and glutaconic and malonic acids were among the compounds identified in trace amounts. Other unidentified compounds were also present, especially in the non-catalytic experiments; but they were detected only in insignificant amounts.

### 2.1. Influence of Temperature and Reaction Time

**Figure 1:**
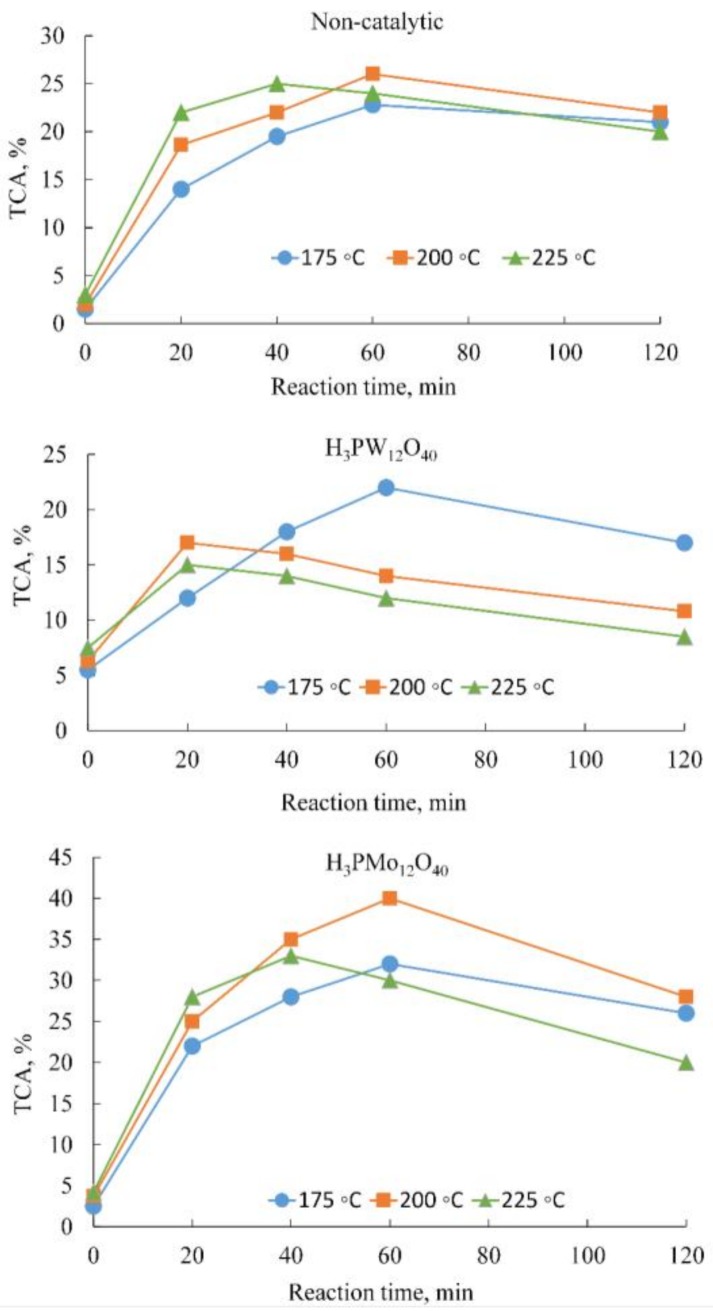
Effect of temperature and reaction time on the total carboxylic acids (TCA) yield. In the catalytic experiments, the lignin:catalyst ratio was 2:1 (*w*/*w*).

To study the effects of temperature and reaction time on the total carboxylic acid yield and lignin conversion, experiments were carried out at 175 °C, 200 °C, and 225 °C. The quantities of reactants used for the non-catalytic experiments were 4 g of lignin and 100 mL of water; and for the catalytic experiments, the reaction mixture contained 4 g of lignin, 100 mL of water and 2 g of catalyst (H_3_PW_12_O_40_ or H_3_PMo_12_O_40_). As shown in Figure 1, both temperature and reaction time played key roles with regard to the yield of carboxylic acids obtained from the partial oxidation of lignin.

As shown in Figure 1, the optimum temperature for maximum yield of products was 200 °C for the non-catalytic and H_3_PMo_12_O_40_ experiments, and 175 °C for the H_3_PW_12_O_40_ catalyst. Sixty minutes was the optimal reaction time in all three cases. At longer reaction times, the products started to decompose. The maximum yield of total carboxylic acids (TCA) obtained was 26% for the non-catalytic experiments, 40% for the phosphomolybdic acid (H_3_PMo_12_O_40_) experiments, and 22% for the phosphotungstic acid (H_3_PW_12_O_40_) experiments. In general, phosphotungstic acid gave the lowest total carboxylic acid yield when compared to the non-catalytic and phosphomolybdic acid experiments. At 175 °C, the H_3_PW_12_O_40_ catalyst gave better yields and selectivity of formic acid than found with the non-catalytic experiments (see Table 1). However, the H_3_PW_12_O_40_ catalyst exhibited less selectivity towards acetic and succinic acids, and consequently gave lower yields of these acids, thus resulting in a lower overall yield of carboxylic acid. Another possible reason for the lower TCA yield with the H_3_PW_12_O_4_ catalyst could be that the produced lower molecular carboxylic acids were further decomposed to CO_2_ and water in the presence of oxygen and catalyst, leading to a reduced overall carboxylic acid yield.

Overall, the H_3_PMo_12_O_40_ catalyst provided the best yields and selectivity for formic and acetic acids in almost all conditions. The H_3_PW_12_O_4_ and non-catalytic experiments showed slightly better succinic acid selectivity than the H_3_PMo_12_O_40_ experiments. This, however, is because both the non-catalytic and H_3_PW_12_O_4_ experiments provided lower lignin conversions relative to the H_3_PMo_12_O_40_ catalyst, hence the selectivity—which is defined here as the yield of each product divided by lignin conversion—becomes higher. On the other hand, all the experiments showed, more or less, comparable yields for succinic acid.

In aqueous solutions, HPAs, such as H_3_PW_12_O_40_, and H_3_PMo_12_O_40_, are strong and fully dissociated acids [38]. The relative activities of Keggin HPAs depend primarily on their acid strengths [38]. A comparison of the dissociation constants and relative acid strengths of the HPAs and mineral acids is shown in Table 2, using the data in [38] for acids in acetone. Table 2 shows that the two studied heteropoly acids are stronger acids than common mineral acids, such as H_2_SO_4_, HCl, and HNO_3_. The tungsten acids are notably stronger than the molybdenum acids (see Table 2). Relative to other heteropoly acids, H_3_PW_12_O_40_ is regarded as the strongest acid in the Keggin series [38]. This characteristic often makes phosphotungstic acid preferable for the acid hydrolysis of biomass [39,40]. Acid hydrolysis, unlike partial oxidation, occurs without the presence of oxidants, such as oxygen.

**Figure 2:**
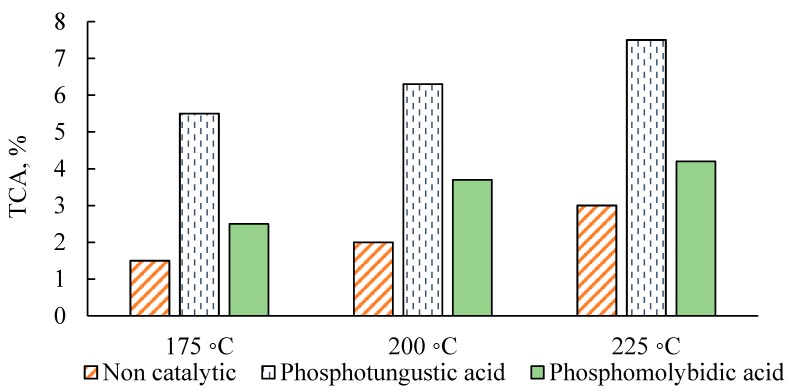
Effect of preheating and acid hydrolysis at 0 reaction time. The lignin:catalyst mass ratio for the catalytic experiments was 2:1 (*w*/*w*).

The effect of HPAs during batch oxidation experiments is a combination of acid hydrolysis and oxidation. The effect of the acid hydrolysis can be analyzed from the samples taken at 0 reaction time, which is the time needed to preheat the samples to the desired temperature before the start of oxidation. As shown in Figure 2, the phosphotungstic acid catalyst gave the maximum yield of carboxylic acids during the preheating period in all cases. This result can be attributed to the excellent acid hydrolyzing ability of phosphotungstic acid, due to its strong acid strength. In addition, the yield of total carboxylic acids during the preheating period greatly increased when the reaction temperature was increased.

Despite the remarkable acid hydrolysis potential of phosphotungstic acid during the preheating period, it seems that the partial oxidation of lignin is less controlled by the acid strength of the catalysts. Rather, it is the oxidation potential of the catalysts that seems to play a pivotal role in the conversion of lignin into products. Having higher oxidation potentials than tungsten HPAs, molybdenum HPAs have superior oxidizing abilities for lignin. The oxidation potential of HPAs, which determines the reducibility of HPAs, follows the order PM_O_ > SiMo >> PW > SiW [38]. Therefore, the higher yield of carboxylic acids achieved by H_3_PMo_12_O_40_ could be attributed to its superior oxidation potential. 

**Figure 3:**
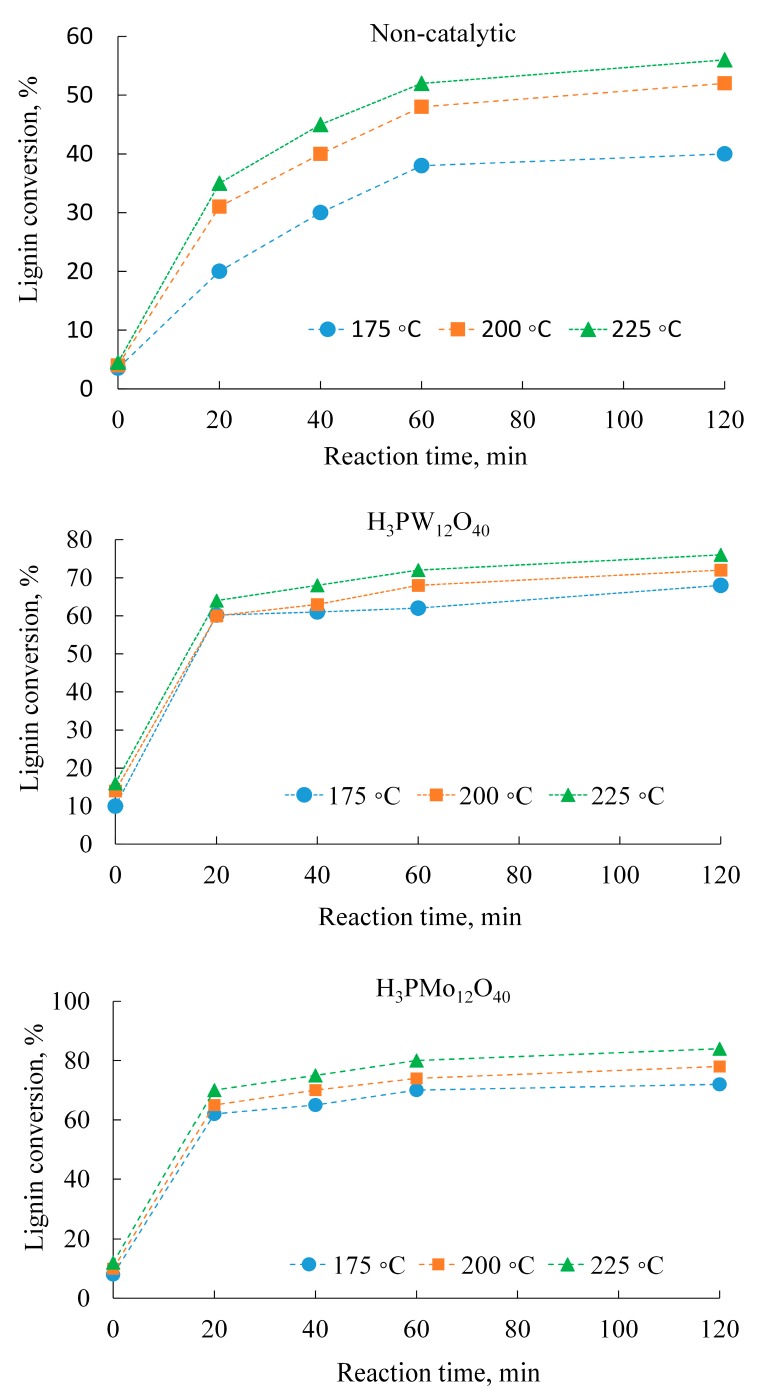
Effects of temperature and reaction time on lignin conversion. In the catalytic experiments, the lignin:catalyst ratio was 2:1 (*w*/*w*).

On the other hand, as shown in Figure 3, both catalysts provided significantly better lignin conversions than the non-catalytic experiments. In all cases, increasing the reaction temperature and reaction time provided higher lignin conversions. At 225 °C and a reaction time of 2 h, the maximum lignin conversations achieved were 56% for the non-catalytic, 76% for the H_3_PW_12_O_40_, and 84% for the H_3_PMo_12_O_40_ experiments. For the catalytic experiments, the lignin conversions were determined at a lignin:catalyst ratio of 2:1. However, both the yield of products and the conversion of lignin were affected by the amount of catalyst used during the partial wet oxidation reaction.

### 2.2. Effect of Catalyst Concentration

**Figure 4:**
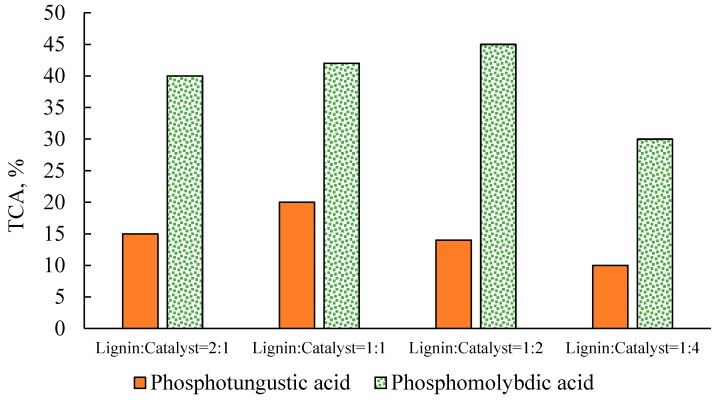
Effect of the lignin to catalyst ratio (*w*:*w*) on the total yield of carboxylic acids at 200 °C with a 60-min reaction time.

The effect of the lignin to catalyst mass ratio was investigated at 200 °C, with a 60-min reaction time. As shown in Figure 4, the total yield of carboxylic acids was significantly affected by the lignin to catalyst mass ratio used. With the H_3_PW_12_O_40_ catalyst, the yield of total carboxylic acids increased from 15 to 20%, when increasing the ratio amount of catalyst from half to equal amounts, relative to lignin. However, further increases in the amount of catalyst, to twice or four times the amount of lignin, resulted in a reduced yield of products. With the H_3_PMo_12_O_40_ catalyst, the yield of carboxylic acids increased slightly, from 40 to 45%, when increasing the amount of catalyst by up to two times the amount of lignin. However, when the concentration of H_3_PMo_12_O_40_ was four times the amount of lignin, the overall carboxylic acid yield was reduced to 30%.

**Figure 5:**
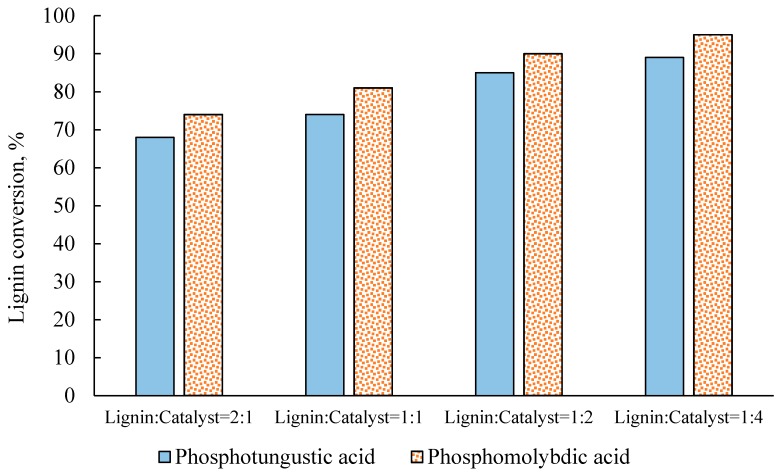
Effects of the lignin to catalyst ratio (*w*:*w*) on lignin conversion at 200 °C, with a 60-min reaction time.

Unlike the yield of products, the lignin conversion steadily increased at higher catalyst concentration levels (see Figure 5). The highest lignin conversion was achieved when the catalyst to lignin mass ratio was 4:1, resulting in a 95% lignin conversion for the H_3_PMo_12_O_40_ catalyst and about 90% for the H_3_PW_12_O_40_ catalyst. The lignin conversions obtained from both catalysts were considerably higher than those obtained from heterogenous catalysts, such as LaMnO_3_ [22] and LaCoO_3_ [41], or base catalysts, described in [42]. However, it is noteworthy that the reaction conditions used in [22,41,42] differed from those in this work. 

### 2.3. Recoverability of the Catalysts

The main bottleneck, limiting the use of homogeneously catalyzed processes, is the difficulties in catalyst recovery and recycling. As the cost of HPAs is higher than that of mineral acids, effective recycling of HPA catalysts is an important issue for the commercial viability of potential applications. Different methods have been considered to recover the used HPA catalysts. As macromolecules, HPAs have been effectively recovered by mesoporous ceramic membrane, without loss of catalytic activity [43]. Solvent extraction has also been used to recover HPA catalysts [44]. However, the feasibility of this technology is questionable for large-scale industrial processes. Another potential method for product and catalyst recovery is, as described in [45], to react the formed carboxylic acids with low molecular alcohols, such as methanol, to generate volatile ester compounds, which can be readily removed from the reaction mixture by evaporation or distillation. The catalyst remaining in the bottom flow can be recirculated back to the reactor.

To demonstrate the recoverability of the used catalysts, additional experiments were carried out at using the following reaction conditions: a lignin:catalyst ratio of 1:1 (*w*/*w*), 175 °C, and a 1 h reaction time. The reaction was stopped by rapidly cooling the reactor in an ice bath to room temperature and removing the oxygen from the reactor. The reaction mixture, which contained an aqueous solution of oxidation products, the catalyst, and fragments of unreacted lignin and/or char, was vacuum-filtered and collected for subsequent catalyst recovery experiments. With the H_3_PW_12_O_40_ catalyst, 50 mL of diethyl ether was added to 25 mL of the filtered solution and the extraction was carried out for about 3 h at room temperature and at a mixing rate of 250 rpm. The resulting mixture was then settled for 2 h. Three layers were formed after the phases had completely separated. The lowest layer was a phosphotungstic acid–ether complex, with a larger specific gravity. The top layer, which predominantly comprised excess diethyl ether, had the lowest specific gravity. The middle layer contained mainly the lignin oxidation products and water. The lowest layer (phosphotungstic acid–ether complex) was then collected and the H_3_PW_12_O_40_ catalyst was recovered, after the removal of diethyl ether by rotary vacuum evaporation, with mild heating on a water bath. More than 82% of the recovery of the catalyst was achieved in just one extraction step. The recovery of the H_3_PMo_12_O_40_ catalyst was carried out by simply distilling the products and water out of the filtered solution in a vacuum rotary dryer at 90 °C, by exploiting the high molecular weight of the H_3_PMo_12_O_40_. Good recovery is possible with both methods, but the economic and technical feasibilities for large scale processes require further study.

## 3. Materials and Methods

### 3.1. Materials

A commercially available alkali lignin purchased from Sigma-Aldrich (Schnelldorf, Germany) was used in the experiments. Deionized water was used in all the experiments. The two heteropoly acids, H_3_PW_12_O_40_ and H_3_PMo_12_O_40_, were bought from Sigma-Aldrich and were used as provided.

### 3.2. Experimental Procedure and Method

The partial wet oxidation experiments were conducted in a batch Parr autoclave reactor, constructed of type 316 stainless steel, with an internal volume of 450 mL. The desired amounts of lignin, catalyst and distilled water were introduced into the reactor, and the reaction mixture was heated to the desired operating condition. The air inside the reactor vessel was removed prior to starting the experiments using 5 bars of nitrogen, to avoid possible oxidation of lignin with air during the preheating period before the desired operating temperature was reached. The reaction mixture was stirred at a constant rate of 450 rpm throughout the experiments. After preheating of the solution to the desired reaction temperature, pure oxygen was introduced into the vessel up to the desired oxygen partial pressure (10 bar). The temperature inside the reactor was kept constant by a water cooling system that was controlled by a solenoid valve. Samples were taken at different time intervals. The samples were vacuum filtered using 0.45 µm filter paper before subsequent analysis. The composition of the wet oxidation products in the liquid samples was analyzed by high-performance liquid chromatography (HPLC). In the HPLC analysis, an eluent of 0.005 M H_2_SO_4_, flow rate 0.6 mL/min, injection volume 10 μL, UV detection (250 nm), refractive index detection (RID) and a MetaCarb 87H (300 × 7.8 mm) column at 35 °C was used. Peak identification of the partially oxidized intermediate compounds from the wet oxidation reaction was done by comparing the sample peak retention times with those of standard solutions of pure compounds. Lignin conversion was determined by the change in lignin mass before and after the reaction. Non-catalytic experiments, using lignin and distilled water as reactants, were performed for comparison with the catalytic experiments.

## 4. Conclusions

The potential of two types of heteropoly acid catalyst for the production of carboxylic acids via partial wet oxidation of lignin was examined at different operational conditions. With the non-catalytic and the H_3_PMo_12_O_40_ experiments, the optimum conditions for maximum yield of products were a temperature of 200 °C and reaction time of 60 min. With the H_3_PW_12_O_40_ experiments, the optimal conditions were 175 °C and a reaction time of 60 min. It was also found that the amount of catalyst used, relative to the lignin, remarkably affected both the yield of the products and the lignin conversion rate. Of the two catalysts studied, the phosphomolybdate catalyst (H_3_PMo_12_O_4_) provided the best results, with a maximum yield of carboxylic acids of up to 45% and lignin conversion of 95%. The two heteropoly acids showed different catalytic behaviors and the reaction pathway of lignin oxidation seems to be determined by the type of addenda atom in the HPA catalyst, with Mo favoring a selective oxidation reaction. Recovery of the catalyst was easily achieved for both HPAs, making partial lignin wet oxidation an environmentally friendly and potentially economically viable process.

## Figures and Tables

**Table 1:** Yields and selectivity of the main detected carboxylic acids at a reaction time of 60 min. The lignin to catalyst ratio was 2:1 (*w*/*w*) for both catalysts.

Experiment	Temperature (°C)	Conversion (%)	TCA (%)	Formic Acid		Acetic Acid		Succinic Acid	
				Yield (%)	Selectivity (%)	Yield (%)	Selectivity (%)	Yield (%)	Selectivity (%)
	175	38	22.5	8.6	22.5	6.1	16.0	0.9	2.4
**Non-catalytic**	200	48	26.0	16.0	33.3	6.5	13.5	1.2	2.5
	225	52	24.0	12.0	23.1	4.8	9.2	0.8	1.5
	175	62	22.0	15.4	24.8	0.4	0.7	1.8	1.8
**H_3_PW_12_O_4_**	200	60	14.0	9.8	16.3	0.3	0.5	1.1	2.8
	225	72	12.0	8.4	11.7	0.2	0.3	1.0	3.8
	175	70	33.0	21.5	30.6	8.9	12.7	1.0	1.4
**H_3_PMo_12_O_40_**	200	74	40.0	26.0	35.1	10.8	14.6	1.2	1.6
	225	80	30.0	19.5	24.4	8.1	10.1	0.9	1.1

**Table 2:** Dissociation constants of heteropoly acids in acetone at 25 °C [38].

Acid	p*K*_1_	p*K*_2_	p*K*_3_
H_3_PW_12_O_40_	1.6	3	4
H_3_PMo_12_O_40_	2	3.6	5.3
H_2_SO_4_	6.6		
HCl	4.3		
HNO_3_	9.4		
