# Erratum: Demesa, A.G.; et al. Valorization of Lignin by Partial Wet Oxidation Using Sustainable Heteropoly Acid Catalysts. *Molecules* 2017, *22*, 1625

**DOI:** 10.3390/molecules23071625

**Published:** 2018-07-04

**Authors:** Abayneh Getachew Demesa, Arto Laari, Mika Sillanpää, Tuomas Koiranen

**Affiliations:** 1 Laboratory of Process and Product Development, LUT School of Engineering Science, Lappeenranta University of Technology, Skinnarilankatu 34, FI-53850 Lappeenranta, Finland; arto.laari@lut.fi (A.L.); tuomas.koiranen@lut.fi (T.K.); 2 Laboratory of Green Chemistry, LUT School of Engineering Science, Lappeenranta University of Technology, Sammonkatu 12, FI-50130 Mikkeli, Finland; mika.sillanpaa@lut.fi

The authors would like to make the following correction to their published paper [1]. We found that a wrong figure was used as Figure 1 in the article. Figure 1 should have been about the ‘effect of temperature and reaction time on the total carboxylic acids (TCA) yield’, as indicated in the caption, but the figure used in the original paper showed the ‘effects of temperature and reaction time on lignin conversion’. However, both the caption and the discussions regarding Figure 1 will remain unchanged. The corrected Figure 1 should be:

The authors would like to apologize for any inconvenience caused to the readers by this change. The change does not affect the scientific results. The manuscript will be updated and the original will remain online on the article webpage, with a reference to this Correction.

## Figures and Tables

**Figure 1:**
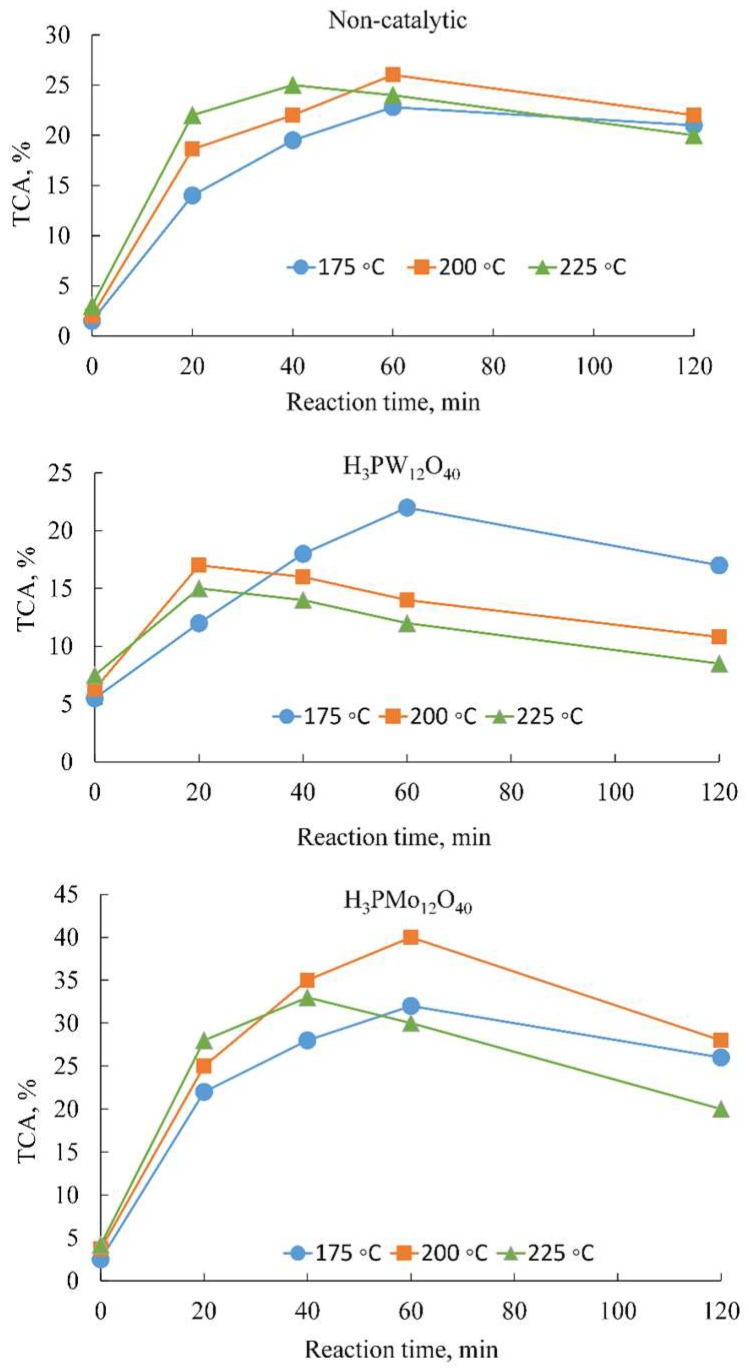
Effect of temperature and reaction time on the total carboxylic acids (TCA) yield. In the catalytic experiments, the lignin:catalyst ratio was 2:1 (*w*/*w*).

## References

[1] Demesa A.G., Laari A., Sillanpää M., Koiranen T. (2017). Valorization of lignin by partial wet oxidation using sustainable heteropoly acid catalysts. Molecules.

